# Smart Sensor Technologies for IoT

**DOI:** 10.3390/s21175890

**Published:** 2021-09-01

**Authors:** Peter Brida, Ondrej Krejcar, Ali Selamat, Attila Kertesz

**Affiliations:** 1 Faculty of Electrical Engineering and Information, University of Zilina, Univerzitna 1, 01026 Zilina, Slovakia; 2 Center for Basic and Applied Research, Faculty of Informatics and Management, University of Hradec Kralove, Rokitanskeho 62, 500 03 Hradec Kralove, Czech Republic; ondrej.krejcar@uhk.cz; 3 Malaysia Japan International Institute of Technology (MJIIT), Universiti Teknologi Malaysia, Jalan Sultan Yahya Petra, Kuala Lumpur 54100, Malaysia; aselamat@utm.my; 4 Department of Software Engineering, University of Szeged, H-6720 Szeged, Hungary; keratt@inf.u-szeged.hu

The recent development in wireless networks and devices leads to novel services that will utilize wireless communication on a new level. It is possible to see a lot of effort and resources invested to establish new communication networks that will support massive machine-to-machine communication and the Internet of Things (IoT). In these systems, various smart and sensory devices are assumed to be deployed and connected, enabling the streaming of large amounts of data.

Smart services represent new trends in mobile services, i.e., a completely new spectrum of context-aware, personalized, and intelligent services and applications. A variety of existing services already utilize information about the position of the user or mobile device. In a lot of applications, the position of mobile devices is achieved thanks to the use of Global Navigation Satellite System (GNSS) chips that are integrated into all modern mobile devices (smartphones). However, GNSS is not always a reliable source of position estimates due to multipath propagation and signal blockage. Moreover, in foreseen IoT applications, the use of GNSS chips integrated into all devices might have a negative impact on their battery life. Therefore, alternative solutions for position estimation should be investigated and implemented in IoT applications. 

Additionally, smart mobile sensors and devices could be able to fulfill an astonishingly wide range of demands of users and providers. One of the reasons behind the wireless device development is the ever-growing computing power together with the reduction of energy consumption and improved communication capabilities of devices. 

In order to process a large amount of data from sensors, further investigation of mobile and dynamic cloud computing solutions is also envisioned. Implementation of new services will be with high probability based on the application of cloud services. This, however, produces additional challenges in such areas as management, security, technical solutions, infrastructure modelling, mobile devices support, and many others.

This Special Issue of Sensors aims at reporting on some of the recent research efforts on this increasingly important topic. The twelve accepted papers in this issue cover various aspects in Smart Sensor Technologies for IoT.

Paper [1] presents the Enhanced Multicast Repair (EM-REP) Fast ReRoute mechanism, which solves several limitations of the legacy M-REP Fast ReRoute mechanism. This means mainly supports for fast reroute in the event of continuous link and node failures throughout the whole network and that the destination host does not have to be directly connected to a network with a router that performs decapsulation, which also reduces the flooding process of M-REP packets in a network with multiple routing areas. The EM-REP mechanism does not require any preparatory calculations, which is effective for IoT devices such as sensors. In the area of WSN and the IoT, it can be used to distribute, for example, urgent messages across the WSN network or to assure the time-critical delivery of important information from sensors to gateways or behind to analytic servers.

In [2], the bit-repair (B-REP) Fast ReRoute mechanism is presented. The proposed mechanism provides advanced fast reroute solutions for IoT and IP network infrastructures. B-REP uses a standardized BIER header with a special bit-string field. That allows to use a standardized header and its fields to define an alternative path as well as to transfer user data. The bit-string, in addition, allows to efficiently define an exact alternative FRR path, which can be calculated by the Dijkstra algorithm or even manually defined by the administrator.

In [3], the new Multilayered Network Model (MNM) for the 5G mobile network’s disrupted infrastructure is introduced. The MNM concept is composed of three independent layers of networks, which are capable of collaboration if disruption of fixed infrastructure occurs. The whole model is able to perform data collection at WSN layer using sensors, which mimic the IoT behaviour by sending those data to the Cloud. If a disruption scenario occurs, only urgent data are allowed to pass into higher layers through the introduced system of WSN gateways. Along with MNM, recommendations for the use of possible wireless technologies with routing protocols are provided. In addition to these recommendations, the exception mechanism for urgent data delivery in routing algorithms is introduced to all layers.

The authors of [4] present a complex IoT-based solution for detecting the position of a lying person. They produced a smart topper based on our novel pressure sensor for measuring the pressure distribution of the lying person. The novel sensor is based on the electrically conductive yarn and the Velostat. The performed experiments indicate a stable resistive response. The functionality of the whole solution and application to the operational staff using the challenging dataset are presented. The modified Convolutional Neural Network classifies the collected data into one of the four lying postures. It achieved an overall accuracy of 82.22% and with the best F1 score of 84%.

The aim of the paper [5] is to compare available sensors from the viewpoint of their suitability for traffic measurements. A summary of the achieved results is given in the form of the score for each sensor. The introduced sensor chart should provide the audience with knowledge about the pros and cons of sensors, especially, if intended for the purposes of road traffic surveillance. The authors in this research focused on the specific situation of road traffic monitoring with magnetometers placed at the roadside. The analysis presented in the paper will serve to the future research when the special sensor node for traffic surveillance will be designed. 

Paper [6] presents the concept and pilot implementation of an indoor tracking system. It is based on an overlapping-resistant Internet of Things (IoT) solution for a Bluetooth Low Energy (BLE)-based indoor tracking system (BLE-ITS). The BLE-ITS is a promising, inexpensive alternative to the well-known GPS. It can be used in human traffic analysis, such as indoor tourist facilities. Tourists or other customers are tagged by a unique MAC address assigned to a simple and energy-saving BLE beacon emitter. Their location is determined by a distributed and scalable network of popular Raspberry Pi microcomputers equipped with BLE and WiFi/Ethernet modules. The authors implemented the prototype and demonstrated its usefulness in a controlled environment.

The growing demand for extensive and reliable structural health monitoring resulted in the development of advanced optical sensing systems that, in conjunction with Wireless Optical Networks (WON), are capable of extending the reach of optical sensing to places where fibre provision is not feasible. To support this effort, the authors of [7] propose a novel MultiWeight Zero Cross-Correlation (MW-ZCC) coding scheme with a low cross-correlation function for Wireless Optical Networks Based Optical Code Division Multiple Access (WON-OCDMA) system. Codes are easy to convert into multiweight power of two codes, thus suitable for supporting a variety of QoS services in WON, including sensing, datacomms and video surveillance applications. The effect of a free space transmission with medium turbulence on the signal transmission and received optical power was analysed. The simulations results revealed that for a minimum allowable BER of 10−3, 10−9, when supporting triple-play services (sensing, datacomms and video surveillance), the proposed WON-OCDMA employing MW-ZCC codes could carry up to 32 services simultaneously at a distance of 32 km in the presence of moderate turbulence in the atmosphere.

In the paper [8], the authors propose an Energy-Efficient Clustering Multi-hop Routing Protocol (EECMR) for routing data packets in Underwater Wireless Sensor Networks (UWSNs). EECMR is a depth-based clustering protocol that uses the depth level of the node to select cluster head nodes and forwarder nodes for multi-hop routing. EECMR considers the residual energy of the node, which elects cluster heads in turns. The nodes can change roles as cluster head, cluster member, and cluster relay. The cluster relay node forwards data from a deeper level to the sink. With the aid of a cluster relay, the energy consumption for transmission is decreased, leading to fewer dead nodes. The simulation results showed that EECMR achieves better performance in terms of higher residual energy, longer network lifetime, and higher received packets at the sink. 

In [9], a new and highly precise system has been presented for electromagnetically scanning large structures. The system combines the range information provided as point clouds by an array of mm-wave radars with the highly accurate positioning data provided by Global Navigation Satellite System-Real-Time Kinematic (GNSS-RTK) modules, forming a sensor-fusion system that enables to merge the point clouds taken from different arbitrary positions. Moreover, communication and control components have been employed to send and receive data from the sensors and manage the system status. As a proof of concept, the system has been tested on a stockpile-like model and in a realistic environment at a seaport with a scaled coal stockpile, obtaining accurate results in both cases.

In [10], the solution for a dynamic radio map collection is introduced. The proposed solution is based on simultaneous measurements of Received Signal Strength (RSS) from Wi-Fi networks and the Inertial Measurement Unit (IMU) data collection. The dead reckoning algorithm processes the IMU data with particle filtering, which helps reduce the localization error of the recovered track. The proposed solution was tested in a real-world environment. The mean localization error of the recovered track was less than 0.6 m, with a maximum error of approximately 2.5 m.

Paper [11] presents a novel human activity recognition system, named WiLiMetaSensing. It realizes location-independent sensing with very few samples in the Wi-Fi environment. Inspired by the idea of meta-learning, the authors endow the system with the ability that can utilize the knowledge acquired from one location for others. The authors proposed a Convolutional Neural Network and Long Short-Term Memory (CNN-LSTM) feature representation and metric learning-based human activity recognition system. The model focuses on the common characteristics of different locations and extracts discriminative features for different activities. The performance evaluation is conducted on the comprehensive dataset built by authors. It demonstrates that the WiLiMetaSensing system can achieve an average accuracy of 91.11%, with four locations for training, given only one sample for other testing locations.

Paper [12] deals with the impact of content on the perceived video quality evaluated using the subjective Absolute Category Rating (ACR) method. The assessment was conducted on eight types of video sequences with diverse content obtained from the SJTU dataset. The sequences were encoded at five different constant bitrates in the two widely used video compression standards H.264/AVC and H.265/HEVC, at Full HD and Ultra HD resolutions, which means 160 annotated video sequences were created. The evaluation was performed in two laboratories: at the University of Zilina, and at the VSB—Technical University in Ostrava. The results acquired in both laboratories showed a high correlation. The evaluation results concluded that it is unnecessary to use the H.265/HEVC codec for compression of Full HD sequences and the compression efficiency of the H.265 codec by the Ultra HD resolution reaches the compression efficiency of both codecs by the Full HD resolution.
